# Neutrophil gelatinase-associated lipocalin prior to cardiac surgery predicts acute kidney injury and mortality

**DOI:** 10.1136/heartjnl-2017-311760

**Published:** 2017-08-09

**Authors:** Heerajnarain Bulluck, Raju Maiti, Bibhas Chakraborty, Luciano Candilio, Tim Clayton, Richard Evans, David P Jenkins, Shyam Kolvekar, Gudrun Kunst, Christopher Laing, Jennifer Nicholas, John Pepper, Derek M Yellon, Derek J Hausenloy

**Affiliations:** 1 National Heart Research Institute Singapore, National Heart Centre, Singapore, Singapore; 2 The Hatter Cardiovascular Institute, University College, London, UK; 3 Papworth Hospital, Cambridge, UK; 4 Centre for Quantitative Medicine Duke-NUS Medical School Academia, Singapore, Singapore; 5 Department of Medical Statistics, London School of Hygiene & Tropical Medicine, London, UK; 6 Barts Heart Centre, St Bartholomew’s Hospital, London, UK; 7 Anaesthetics, Intensive Care Medicine and Perioperative Pain Medicine, King’s College Hospital and King’s College London, London, UK; 8 The Royal Free Hospital, London, UK; 9 National Institute of Health Research Cardiovascular Biomedical Research Unit at Royal Brompton & Harefield National Health Service Trust, London, UK; 10 The National Institute of Health Research, University College London Hospitals, Biomedical Research Centre, London, UK; 11 Cardiovascular and Metabolic Disorders Program, Duke-National University of Singapore Medical School, Singapore, Singapore; 12 Yong Loo Lin School of Medicine, National University Singapore, Singapore, Singapore

**Keywords:** coronary artery disease, coronary artery disease surgery

## Abstract

**Objective:**

We aimed to investigate whether preoperative serum neutrophil gelatinase-associated lipocalin (sNGAL_pre-op_) predicted postoperative acute kidney injury (AKI) during hospitalisation and 1-year cardiovascular and all-cause mortality following adult cardiac surgery.

**Methods:**

This study was a post hoc analysis of the Effect of Remote Ischemic Preconditioning on Clinical Outcomes in Patient Undergoing Coronary Artery Bypass Graft Surgery trial involving adult patients undergoing coronary artery bypass graft. Postoperative AKI within 72 hours was defined using the International Kidney Disease: Improving Global Outcomes classification.

**Results:**

1371 out of 1612 patients had data on sNGAL_pre-op_. The overall 1-year cardiovascular and all-cause mortality was 5.2% (71/1371) and 7.7% (105/1371), respectively. There was an observed increase in the incidence of AKI from the first to the third tertile of sNGAL_pre-op_ (30.5%, 41.5% and 45.9%, respectively, p<0.001). There was also an increase in both cardiovascular and all-cause mortality from the first to the third tertile of sNGAL_pre-op_, linear trend test with adjusted p=0.018 and p=0.013, respectively. The adjusted HRs for those in the second and third tertiles of sNGAL_pre-op_ compared with the first tertile were 1.60 (95% CI 0.78 to 3.25) and 2.22 (95% CI 1.13 to 4.35) for cardiovascular mortality, and 1.25 (95% CI 0.71 to 2.22) and 1.91 (95% CI 1.13 to 3.25) for all-cause mortality at 1 year.

**Conclusion:**

In a cohort of high-risk adult patients undergoing cardiac surgery, there was an increase in postoperative AKI and 1-year mortality from the first to the third tertile of preoperative serum NGAL. Those in the last tertile (>220 ng/L) had an estimated twofold increase risk of cardiovascular and all-cause mortality at 1 year.

**Clinical trial registration:**

NCT101247545; Post-results.

## Introduction

Acute kidney injury (AKI) occurs in up to 30%^1 2^ of adult patients undergoing cardiac surgery, and its presence is associated with worse short-term and long-term morbidity and mortality.^3–5^ Even mild to moderate increases in serum creatinine following cardiac surgery are associated with higher 30-day mortality.^6 7^ Around a fifth of patients with AKI will go on to require renal replacement therapy,^8^ and this subgroup has the worst prognosis with in-hospital mortality rates up to 60%.^9^ The development of AKI is multifactorial, and the underlying mechanisms remain unclear, although acute tubular necrosis is assumed to be the predominant pathology.^10^ Although several tools have been developed to predict the development of AKI prior to cardiac surgery, they have been predominantly designed to predict severe AKI requiring dialysis.^2^ More work remains to be done to improve the identification of those at risk of even milder grades of AKI prior to cardiac surgery.^2^

Neutrophil gelatinase-associated lipocalin (NGAL) belongs to the lipocalin family and is produced predominantly by the liver and white blood cells.^11^ It is released excessively in the blood pool and in the urine after injury to the kidney tubules.^12^ NGAL has previously been termed the ‘troponin-like’ biomarker to detect AKI,^13^ but after promising results in children undergoing cardiac surgery,^14^ it has not performed as well for the early detection of AKI in adults.^15^ This can be partly explained by the fact that NGAL is not specific for the kidney injury, as it can also be released during sepsis, inflammation and acute exacerbations of chronic illnesses, and therefore it remains a research tool.^14^ Most recently, preoperative plasma NGAL has been shown to be independently associated with 3-year mortality following adult cardiac surgery.^16^ There are some conflicting reports on whether preoperative plasma NGAL can predict postoperative AKI,^17 18^ but those studies were underpowered. Therefore, we aimed to investigate whether preoperative serum NGAL (sNGAL_pre-op_) predicted both postoperative AKI during hospitalisation and 1-year cardiovascular and all-cause mortality in adults undergoing cardiac surgery.

## Methods

The current study was a post hoc analysis of the Effect of Remote Ischemic Preconditioning on Clinical Outcomes in Patient Undergoing Coronary Artery Bypass Graft Surgery (ERICCA) (NCT01247545), the trial design and results of which have been previously published.^19 20^ In brief, 1612 patients were prospectively recruited in this multicentre, randomised controlled trial between April 2011 and March 2014 but only those with data on sNGAL_pre-op_ were included in this analysis (n=1371). The inclusion criteria were adults aged >18 years of age and with an additive European System for Cardiac Operative Risk Evaluation (EuroSCORE) ≥5 (high risk), undergoing on-pump coronary artery bypass grafting (CABG) with or without valve surgery, with blood cardioplegia. Those with an estimated glomerular filtration rate (eGFR) <30 mL/min/1.73 m^2^ were excluded. They were randomised to remote ischaemic conditioning (RIC) or sham preconditioning. The clinical endpoints collected were cardiovascular death and all-cause mortality at 1 year, non-fatal myocardial infarction, coronary revascularisation or stroke at 1 year and AKI within 72 hours followingsurgery as defined by the International Kidney Disease: Improving Global Outcomes classification (KDIGO).^21^ There was no difference in the incidence of AKI between those who were randomised to RIC or sham. Serum NGAL was measured from blood collected in serum-separating tubes preoperatively using CircuLex NGAL/Lipocalin 2 ELISA. The study was approved by the National Health Service Research Ethics Committee, and all patients provided informed written consent.

AKI within 72 hours was graded as none, 1, 2, 3 as per the KDIGO criteria as follows: grade 1: serum creatinine 1.5–1.9 times baseline or an increase in serum creatinine ≥26.5 μmol/L; grade 2: serum creatinine 2.0–2.9 times baseline; and grade 3: serum creatinine 3.0 times baseline or increase in serum creatinine to ≥353.6 μmol/L or initiation of renal replacement therapy. The primary outcomes of interest were any AKI grade during hospitalisation and cardiovascular and all-cause mortality at 1 year.

### Statistical analysis

Statistical analysis was performed using SPSS V.23. sNGAL_pre-op_ was analysed as tertiles. Continuous data were expressed as means and SD or medians and (first quartile–third quartile), and categorical data were reported as frequencies and percentages. Unadjusted associations of the tertiles of sNGAL_pre-op_ with other baseline and procedure variables were performed using one-way analysis of variance or Kruskal-Wallis test where appropriate. Logistic regression analysis was performed to assess whether sNGAL_pre-op_ was an independent predictor any grade of AKI (as a binary outcome), after adjusting for known preoperative predictors of AKI (age, gender, diabetes mellitus, hypertension, peripheral vascular disease (PVD), previous CABG, type of surgery planned, use of intra-aortic balloon pump, baseline eGFR, baseline left ventricular ejection fraction (LVEF) category and preoperative high sensitivity troponin T).^2 22^ This analysis was performed using tertiles of sNGAL_pre-op_, as previously done,^16^ to allow findings to be more easily translated to the clinical setting. Kaplan-Meier curves were used to assess survival at 1 year per tertile. The primary analysis for the cumulative incidence of cardiovascular and all-cause mortality at 1 year was performed using Cox proportional hazard (with censoring of data to the date of occurrence of the primary endpoint, lost to follow-up, withdrawal from the study or at 1 year), and the HRs were computed with 95% CI after adjusting for the factors that were present in both EuroSCORE II^23^ and the Society of Thoracic Surgeon (STS) score^24^ that were available from our cohort (namely age, gender, eGFR, PVD, previous stroke, previous CABG, baseline LVEF category, diabetes mellitus, use of intra-aortic balloon pump, type of surgery planned and preoperative high sensitivity troponin T).

## Results

Out of 1612 patients recruited in the ERICCA trial, 1371 patients had sNGAL_pre-op_ measured. These patients had a mean age of 76.0±6.6 years and 27.5% were female. As expected, they were in the high-risk category for cardiac surgery with a median additive EuroSCORE of 6 (5–7). Further details of the baseline characteristics of these 1371 patients per tertile are provided in table 1. The overall all-cause mortality and cardiovascular mortality was 7.7% (105/1371) and 5.2% (71/1371), respectively. AKI data were available in 97% (1330/1371) of patients. AKI during the initial hospitalisation occurred in 39.6% of patients (31.2% AKI grade 1). The median sNGAL_pre-op_ was 175 (117–257) ng/L and range of 27–3171 ng/L. sNGAL_pre-op_ was divided into three groups according to tertiles <135 ng/L, 135–<220 ng/L and ≥220 ng/L. There was no difference in the prevalence of diabetes mellitus, hypertension, dyslipidaemia and vascular disease among the three tertiles as shown in table 1. Furthermore, there was no difference in the type of surgery or duration of the cardiopulmonary bypass and cross-clamp times. However, patients in the higher tertiles were more likely to be older, of the male gender, have higher baseline creatinine and eGFR and spend longer time on intensive care unit and in hospital. The cardiovascular and all-cause mortality at 1 year per tertile and per the presence or absence of AKI are summarised in table 2.

**Table 1:** Characteristics of patients according to tertiles of sNGAL_pre-op_

n=1371	sNGAL_pre-op_			p Value
	First tertile <135 ng/L (n=457)	Second tertile 135 –<220 ng/L (n=456)	Third tertile >220 ng/L (n=458)	
Age/years	75.4±7.0	76.1±6.1	76.6±6.6	0.03
Male (n (%))	302 (66.2)	342 (75.3)	345 (75.8)	0.001
Additive EuroSCORE	6 (5–7)	6 (5–7)	6 (5–8)	0.03
BMI/kg/m^2^	27.2±4.5	27.8±4.5	27.7±4.4	0.08
Current smoker (n (%))	26 (5.7)	28 (6.2)	24 (5.3)	0.45
Diabetes mellitus (n (%))	112 (24.6)	119 (26.2)	124 (27.3)	0.65
Dyslipidaemia (n (%))	321 (70.4)	331 (72.9)	316 (69.5)	0.50
Hypertension (n (%))	345 (75.7)	340 (74.9)	353 (77.6)	0.62
Previous MI (n (%))	166 (36.4)	189 (41.6)	197 (43.3)	0.09
Atrial fibrillation (n (%))	70 (15.4)	71 (15.6)	82 (18.0)	0.49
PVD (n (%))	40 (8.8)	32 (7.0)	37 (8.1)	0.63
Stroke	51 (11.2)	53 (11.7)	51 (11.2)	0.97
LVEF category (n (%))				0.55
Good	300 (68.8)	296 (67.6)	276 (63.6)	
Moderate	89 (20.4)	93 (21.2)	103 (23.7)	
Poor	47 (10.8)	49 (11.2)	55 (12.7)	
Baseline creatinine/µmol/L	83 (73–97)	91 (76–108)	95 (80–118)	<0.001
eGFR at baseline/mL/min/1.73 m^2^	75 (62–90)	71 (59–85)	67 (53–82)	<0.001
Cardiopulmonary bypass time/hour	1.75 (1.39–2.20)	1.72 (1.33–2.23)	1.77 (1.35–2.35)	0.34
Cross-clamp time/hour	1.17 (0.80–1.55)	1.12 (0.78–1.57)	1.15 (0.83–1.63)	0.26
CABG only (n (%))	229 (50.2)	243 (53.3)	228 (50.1)	0.55
CABG+valve surgery (n (%))	227 (49.8)	213 (46.7)	227 (49.9)	
Length of ITU stay	2 (1–4)	3 (1–5)	3 (1–5)	<0.001
Length of hospital stay	9 (7–14)	10 (7–16)	12 (8–21)	<0.001
AKI (any) (n (%))	135 (30.5)	186 (41.5)	206 (45.9)	<0.001
PMI	112 (24.5)	102 (22.4)	118 (25.8)	0.48
Stroke	11 (2.4)	5 (1.1)	7 (1.5)	0.29
Revascularisation	1 (0.2)	3 (0.7)	2 (0.4)	0.60

AKI, acute kidney injury; BMI, body mass index; CABG, coronary artery bypass graft surgery; eGFR, estimated glomerular filtration rate; EuroSCORE, European System for Cardiac Operative Risk Evaluation; ITU, intensive care unit; LVEF, left ventricular ejection fraction; MI, myocardial infarction; PMI, perioperative myocardial infarction; PVD, peripheral vascular disease; RIC, remote ischaemic conditioning; sNGAL_pre-op_, preoperative serum neutrophil gelatinase-associated lipocalin.

**Table 2:** One-year cardiovascular and all-cause mortality rates per tertiles of sNGAL_pre-op_ and per presence or absence of AKI

	First Tertile <135 ng/L (n=457)	Second Tertile 135 –<220 ng/L n=456	Third Tertile >220 ng/L n=458	Adjusted p value	No AKI (n=803)	AKI (n=527)	Adjusted p value
1-year cardiovascular mortality	3.5% (16)	5.3% (24)	6.8% (31)	0.018	2.7% (22)	7.8% (41)	<0.001
1-year all-cause mortality	5.5% (25)	7.0% (32)	10.5% (48)	0.013	4.1% (33)	12.1% (64)	<0.001

AKI, acute kidney injury; sNGAL_pre-op_, preoperative serum neutrophil gelatinase-associated lipocalin.

### sNGAL_pre-op_ and AKI

On a per-tertile basis, there was an increase in the incidence of AKI with increasing tertile of sNGAL_pre-op_ from 30.5% in the first tertile, 41.5% in the second tertile and 45.9% in the third tertile. Using the first tertile of sNGAL_pre-op_ as reference, multivariable analysis shown that the second tertile of sNGAL_pre-op_ had an OR of 1.69 (95% CI 1.23 to 2.30), and the third tertile of sNGAL_pre-op_ had an OR of 1.53 (95% CI 1.12 to 2.10), p=0.009 for linear trend test, to predict AKI, after adjusting for known preoperative predictors of AKI.

**Figure 1:**
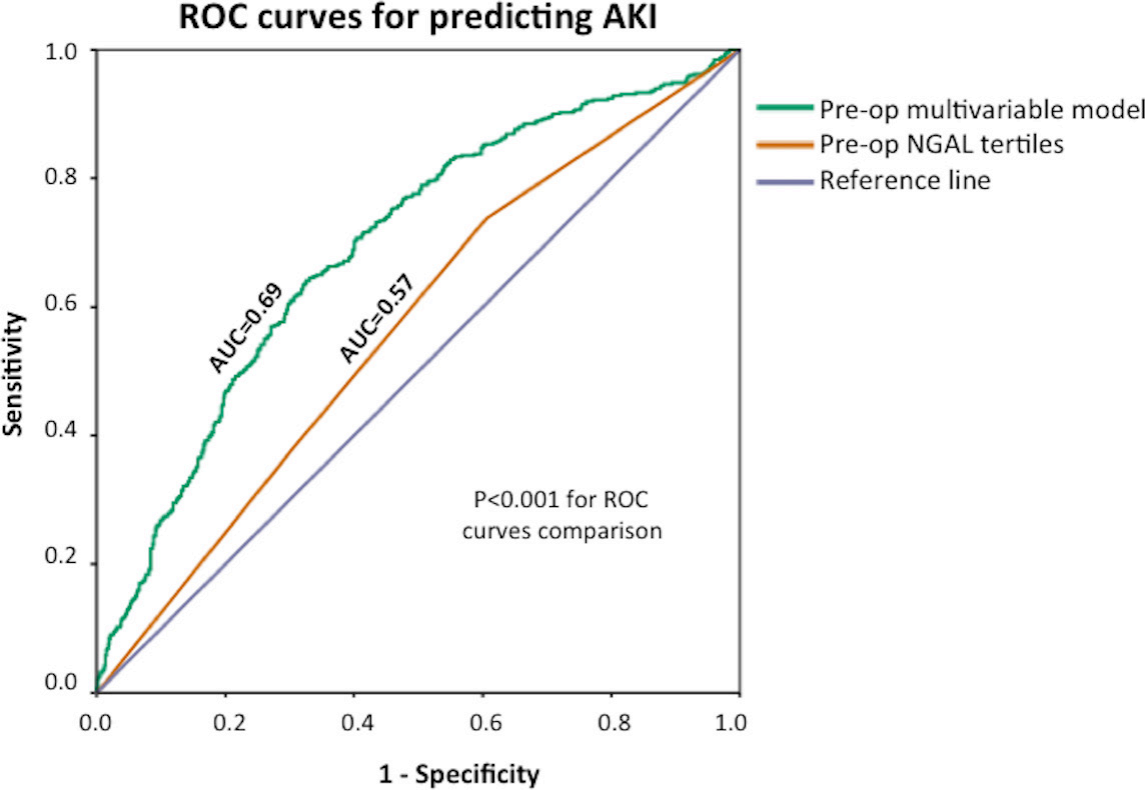
ROC curves of sNGAL_pre-op_ in tertiles and the preoperative multivariable model to predict AKI. AKI, acute kidney injury; AUC, area under the curve; ROC, receiver operating characteristic; sNGAL_pre-op_, preoperative serum neutrophil gelatinase-associated lipocalin.

Receiver operating characteristic (ROC) curve analysis showed that sNGAL_pre-op_ was a predictor of AKI, but the c-statistic was only 0.57 (95% CI 0.54 to 0.61). The c-statistic improved to 0.69 (95% CI 0.66 to 0.73) (p<0.001 for ROC curves comparison) when age, gender, diabetes mellitus, hypertension, PVD, previous CABG, type of surgery planned, use of intra-aortic balloon pump, baseline eGFR, baseline ejection fraction and baseline high sensitivity troponin T were included in the model together with tertiles of sNGAL_pre-op_ as shown in figure 1.

### AKI and mortality

Patients with AKI had a higher mortality at 1 year with an all-cause mortality rate of 12.1% versus 4.1% (table 2), when compared with those without AKI with an adjusted HR (adjusted for the above EuroSCORE II and STS-derived preoperative predictors of mortality) of 2.67 (95% CI 1.72 to 4.13), adjusted p<0.001 from the Cox regression analysis. Those with AKI also had worse cardiovascular mortality at 1 year with a mortality rate of 7.8% versus 2.7%, when compared with those without AKI, with an adjusted HR of 2.88 (95% CI 1.67 to 4.98), adjusted p<0.001 from the Cox regression analysis.

### sNGAL_pre-op_ and mortality

**Figure 2:**
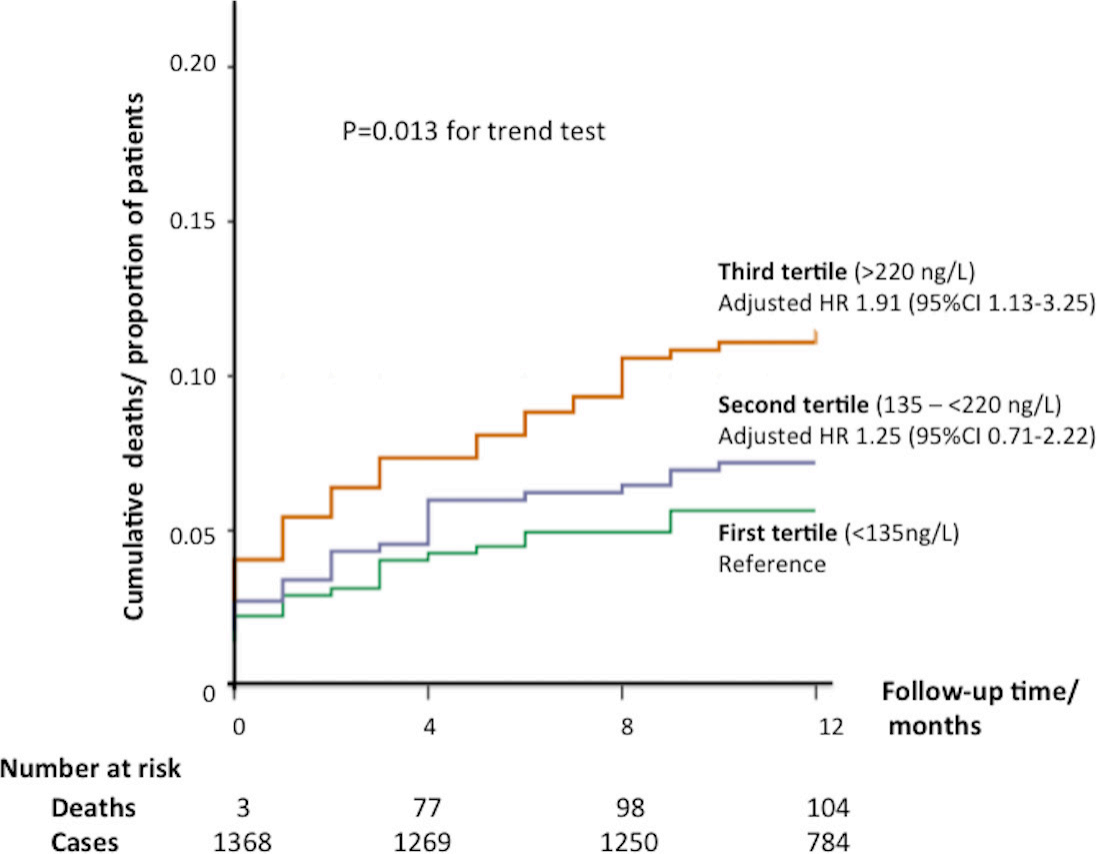
Kaplan-Meier curves for 1-year all-cause mortality according to tertiles of sNGAL_pre-op_. sNGAL_pre-op_, preoperative serum neutrophil gelatinase-associated lipocalin.

After a follow-up of 12 months, 5.5% of patients in the first tertile died, compared with 7.0% in the second tertile and 10.5% of the patients in the third tertile as summarised in table 2. There was an increase in both cardiovascular and all-cause mortality from the first to the third tertile of sNGAL_pre-op_, linear trend test with adjusted p=0.018 and p=0.013, respectively. With sNGAL_pre-op_ in the first tertile as the reference, the adjusted HR (adjusted for the above EuroSCORE II and STS-derived preoperative predictors of mortality) for those in the second and third tertiles of sNGAL_pre-op_ were 1.60 (95% CI 0.78 to 3.25) and 2.22 (95% CI 1.13 to 4.35) for cardiovascular mortality, and 1.25 (95% CI 0.71 to 2.22) and 1.91 (95% CI 1.13 to 3.25) for all-cause mortality at 1 year. Figure 2 shows the Kaplan-Meier curves for 1-year all-cause mortality according to tertiles of sNGAL_pre-op_.

## Discussion

The major findings of this study of high-risk patients undergoing cardiac surgery were as follows: (1) preoperative serum levels of NGAL predicted postoperative AKI, after accounting for preoperative clinical parameters (age, gender, diabetes mellitus, hypertension, PVD, previous CABG, type of surgery planned, use of intra-aortic balloon pump, baseline eGFR, baseline ejection fraction and baseline high sensitivity troponin T), with the performance of sNGAL_pre-op_ to predict AKI improving when these clinical parameters were included in the model; (2) tertiles of sNGAL_pre-op_ were associated with mortality after adjusting for confounders, with those in the third tertile having a twofold increase in the hazard for cardiovascular and all-cause mortality; (3) the overall incidence of postoperative AKI in this cohort was 39.6% and was as high as 45.9% for those in the third tertile of sNGAL_pre-op_; (4) the presence of AKI was associated with mortality, and in our cohort, there was more than twofold increase in both cardiovascular and all-cause mortality at 1 year.

The performance of the model to predict AKI was improved when clinical parameters were included in addition to sNGAL_pre-op_, but the c-statistic was only increased to 0.69. Other risk scores to predict AKI have had c-statistics ranging between 0.72 and 0.84, but most of them were designed to predict worse grades of AKI.^2^ Therefore, Huen *et al* 2 recently concluded that more studies are required to develop risk models to predict milder forms of AKI after cardiac surgery in a systematic review of existing risk scores. However, our study included high-risk patients with EuroSCORE ≥5 only and whether by applying the same approach of using sNGAL_pre-op_ with clinical parameters to predict any grades of AKI would yield a higher diagnostic performance warrants further investigation.

Postoperative urinary NGAL^25^ in contrast to postoperative plasma NGAL^16^ has been shown to be associated with all-cause 3-year mortality. Whether pNGAL_pre-op_ could predict AKI and mortality has also been investigated. Haase-Fielitz *et al* 17 found no difference in preoperative serum NGAL between those who developed AKI when compared with those who did not. However, this study only included 100 patients and did not have mortality data. However, Doi *et al* 18 showed that preoperative plasma NGAL was an independent risk factor for postoperative AKI in 146 patients. A larger study of 1191 patients showed that preoperative plasma NGAL was a predictor of 3-year mortality, but the relationship between NGAL and AKI was not assessed.^16^ In the general population, baseline plasma NGAL has also been shown to be associated with 10-year cardiovascular mortality.^26^ In a cohort of community-dwellers with a mean age of 70 years, plasma NGAL was a significant predictor of mortality, independent of traditional risk factors and renal function.^27^ We have shown in a larger cohort of high-risk patients that sNGAL_pre-op_ was a predictor of both AKI following cardiac surgery, as well as a predictor of 1-year cardiovascular and all-cause mortality, after adjusting for known preoperative predictors of AKI. Therefore, sNGAL_pre-op_ could complement existing tools to risk-stratify patients prior to cardiac surgery.

AKI has typically been reported to occur in up to 30%^1 2^ of patients undergoing adult cardiac surgery, but the incidence of AKI in our cohort was higher at 39.6%. This may be explained by the inclusion of patients with an additive EuroSCORE of ≥5 and therefore representative of the incidence of AKI in a high-risk cohort.

### Limitations

Our cohort may not be representative of the wider population of patients undergoing CABG surgery as these patients were included in a randomised controlled trial. There are currently no established cut-off values as shown by the study by Moledina *et al*,^16^ which used cut-off values of 155 ng/L and 251 ng/L for the tertiles and we used 130 ng/L and 220 ng/L. Although intraoperative factors play a role in the development of postoperative AKI, we only looked at preoperative factors as the aim of this study was to assess the ability of sNGAL_pre-op_ to identify those at higher risk of AKI and mortality prior to surgery. Ten per cent of the cases had missing data when it came to performing the analysis for the adjusted models. We did not specifically collect data on sepsis or exacerbation of chronic illness prior to surgery, which are known confounders for elevated NGAL levels. However, these patients were considered fit enough for major heart surgery, and therefore it is unlikely these confounders were present at the time.

## Conclusions

In a cohort of high-risk adult patients undergoing cardiac surgery, preoperative serum NGAL was an independent predictor of postoperative AKI and 1-year cardiovascular and all-cause mortality. Those in the third tertile of preoperative serum NGAL_pre-op_ (>220 ng/L) had a twofold increase risk of postoperative AKI and mortality at 1 year. The risk stratification of patients prior to cardiac surgery may be improved by adding preoperative serum NGAL to existing risk scores for AKI and mortality and warrants further investigations.

**Key messages**

**What is already known about this subject?**

The development of acute kidney injury following adult cardiac surgery is associated with short-term and long-term morbidity and mortality.

**What are the new findings?**

Preoperative serum neutrophil gelatinase-associated lipocalin was an independent predictor of postoperative acute kidney injury and 1-year mortality.

**How might these results change the focus of research or clinical practice?**

Preoperative serum neutrophil gelatinase-associated lipocalin could be used on top of existing risk scores to further risk-stratify those at higher risk of postoperative acute kidney injury and 1-year mortality.

## References

[1] Thiele RH, Isbell JM, Rosner MH, et al AKI associated with cardiac surgery. Clin J Am Soc Nephrol 2015;10:500–14. 10.2215/CJN.07830814 25376763 PMC4348689

[2] Huen SC, Parikh CR Predicting acute kidney injury after cardiac surgery: a systematic review, 2012 1552-6259 (Electronic). 10.1016/j.athoracsur.2011.09.010 PMC3286599 22186469

[3] Brown JR, Cochran RP, Dacey LJ, et al Northern New England Cardiovascular Disease Study Group. Perioperative increases in serum creatinine are predictive of increased 90-day mortality after coronary artery bypass graft surgery. Circulation 2006;114:I-409–0. 10.1161/CIRCULATIONAHA.105.000596 16820609

[4] Koyner JL, Bennett MR, Worcester EM, et al Urinary cystatin C as an early biomarker of acute kidney injury following adult cardiothoracic surgery. Kidney Int 2008;74:1059–69. 10.1038/ki.2008.341 18650797 PMC2745082

[5] Hobson CE, Yavas S, Segal MS, et al Acute kidney injury is associated with increased long-term mortality after cardiothoracic surgery. Circulation 2009;119:2444–53. 10.1161/CIRCULATIONAHA.108.800011 19398670

[6] Lassnigg A, Schmidlin D, Mouhieddine M, et al Minimal changes of serum creatinine predict prognosis in patients after cardiothoracic surgery: a prospective cohort study. J Am Soc Nephrol 2004;15:1597–605. 10.1097/01.ASN.0000130340.93930.DD 15153571

[7] Lok CE, Austin PC, Wang H, et al Impact of renal insufficiency on short- and long-term outcomes after cardiac surgery. Am Heart J 2004;148:430–8. 10.1016/j.ahj.2003.12.042 15389229

[8] Chertow GM, Lazarus JM, Christiansen CL, et al Preoperative renal risk stratification. Circulation 1997;95:878–84. 10.1161/01.CIR.95.4.878 9054745

[9] Bhatt GC, Das RR Early versus late initiation of renal replacement therapy in patients with acute kidney injury-a systematic review & meta-analysis of randomized controlled trials. BMC Nephrol 2017;18:78 10.1186/s12882-017-0486-9 28245793 PMC5331682

[10] Basile DP, Anderson MD, Sutton TA Pathophysiology of acute kidney injury. Compr Physiol 2012;2:1303–53. 10.1002/cphy.c110041 23798302 PMC3919808

[11] Hvidberg V, Jacobsen C, Strong RK, et al The endocytic receptor megalin binds the iron transporting neutrophil-gelatinase-associated lipocalin with high affinity and mediates its cellular uptake. FEBS Lett 2005;579:773–7. 10.1016/j.febslet.2004.12.031 15670845

[12] Haase M, Bellomo R, Devarajan P, et al Accuracy of neutrophil gelatinase-associated lipocalin (NGAL) in diagnosis and prognosis in acute kidney injury: a systematic review and meta-analysis. Am J Kidney Dis 2009;54:1012–24. 10.1053/j.ajkd.2009.07.020 19850388

[13] Devarajan P Review: neutrophil gelatinase-associated lipocalin: a troponin-like biomarker for human acute kidney injury. Nephrology 2010;15:419–28. 10.1111/j.1440-1797.2010.01317.x 20609093

[14] Mishra J, Mori K, Ma Q, et al Neutrophil gelatinase-associated lipocalin: a novel early urinary biomarker for cisplatin nephrotoxicity. Am J Nephrol 2004;24:307–15. 10.1159/000078452 15148457

[15] Mårtensson J, Bellomo R The rise and fall of NGAL in acute kidney injury. Blood Purif 2014;37:304–10. 10.1159/000364937 25170751

[16] Moledina DG, Parikh CR, Garg AX, et al TRIBE-AKI Consortium. Association of Perioperative plasma neutrophil Gelatinase-Associated lipocalin levels with 3-Year mortality after cardiac surgery: a prospective Observational Cohort Study. PLoS One 2015;10:e0129619 10.1371/journal.pone.0129619 26053382 PMC4460181

[17] Haase-Fielitz A, Bellomo R, Devarajan P, et al Novel and conventional serum biomarkers predicting acute kidney injury in adult cardiac surgery--a prospective cohort study. Crit Care Med 2009;37:553–60. 10.1097/CCM.0b013e318195846e 19114878

[18] Doi K, Urata M, Katagiri D, et al Plasma neutrophil gelatinase-associated lipocalin in acute kidney injury superimposed on chronic kidney disease after cardiac surgery: a multicenter prospective study. Crit Care 2013;17:R270 10.1186/cc13104 24215663 PMC4056897

[19] Hausenloy DJ, Candilio L, Laing C, et al Effect of remote ischemic preconditioning on clinical outcomes in patients undergoing coronary artery bypass graft surgery (ERICCA): rationale and study design of a multi-centre randomized double-blinded controlled clinical trial. Clin Res Cardiol 2012;101:339–48. 10.1007/s00392-011-0397-x 22186969

[20] Hausenloy DJ, Candilio L, Evans R, et al Remote ischemic preconditioning and outcomes of cardiac surgery. N Engl J Med 2015;373:1408–17. 10.1056/NEJMoa1413534 26436207

[21] James M, Bouchard J, Ho J, et al Canadian society of Nephrology commentary on the 2012 KDIGO clinical practice guideline for acute kidney injury. Am J Kidney Dis 2013;61:673–85. 10.1053/j.ajkd.2013.02.350 23518195

[22] Brown JR, Cochran RP, Leavitt BJ, et al Multivariable prediction of renal insufficiency developing after cardiac surgery. Circulation 2007;116:I-139–0. 10.1161/CIRCULATIONAHA.106.677070 17846294

[23] Nashef SA, Roques F, Sharples LD, et al EuroSCORE II. (1873-734X).

[24] Anderson RP First publications from the Society of thoracic Surgeons National Database. Ann Thorac Surg 1994;57:6–7. 10.1016/0003-4975(94)90355-7 8279920

[25] Coca SG, Garg AX, Thiessen-Philbrook H, et al Urinary biomarkers of AKI and mortality 3 years after cardiac surgery. J Am Soc Nephrol 2014;25:1063–71. 10.1681/ASN.2013070742 24357673 PMC4005309

[26] Lindberg S, Jensen JS, Mogelvang R, et al Plasma neutrophil gelatinase-associated lipocalinin in the general population: association with inflammation and prognosis. Arterioscler Thromb Vasc Biol 2014;34:2135–42. 10.1161/ATVBAHA.114.303950 24969771

[27] Daniels LB, Barrett-Connor E, Clopton P, et al Plasma neutrophil gelatinase-associated lipocalin is independently associated with cardiovascular disease and mortality in community-dwelling older adults: the Rancho Bernardo Study. J Am Coll Cardiol 2012;59:1101–9. 10.1016/j.jacc.2011.11.046 22421304 PMC3312791

